# Multiplex Real-Time Polymerase Chain Reaction for Simultaneous Quantification of* Salmonella *spp.,* Escherichia coli*, and* Staphylococcus aureus* in Different Food Matrices: Advantages and Disadvantages

**DOI:** 10.1155/2018/6104015

**Published:** 2018-09-25

**Authors:** Amanda Teixeira Sampaio Lopes, George Rêgo Albuquerque, Bianca Mendes Maciel

**Affiliations:** ^1^Graduation Program in Animal Science, Santa Cruz State University, Ilhéus (BA), Brazil; ^2^Department of Agricultural and Environmental Sciences, Santa Cruz State University, Ilhéus (BA), Brazil; ^3^Department of Biological Sciences, Santa Cruz State University, Ilhéus (BA), Brazil

## Abstract

Quantitative real-time polymerase chain reactions (qPCRs) of the most prevalent bacteria causing foodborne diseases worldwide, such as* Salmonella *spp.,* Escherichia coli*, and* Staphylococcus aureus,* can be an important tool for quantitative microbial risk assessment, which requires numerical data to determine the level of contamination at a specific stage of food production. However, most of qPCR assays described in the literature for these pathogens are qualitative; their objective is pathogen detection and not pathogen quantification. Thus, the aim of our work was to develop a qPCR for the simultaneous quantification of* Salmonella *spp.,* E. coli*, and* S. aureus *and to propose its use in the analysis of foods, as a tool for microbiological quality monitoring. For this, a multiplex qPCR was standardized for the simultaneous quantification of specific fragments of target genes (*ssf*,* pho*A, and* nuc*) corresponding to each one of the mentioned bacteria. The limit of detection of the technique was 13, 10, and 12 gene copies for* ssf*,* pho*A, and* nuc*, respectively; standard curves showed R^2^ > 0.99, with efficiencies ranging from 99 to 110%, and inter- and intraexperiment reproducibility presented a low coefficient of variation in all trials. This methodology was applied in different food matrices (milk, ground beef, and oyster meat), and the results were compared with official microbiological culture methodology and with ready-to-use test. Advantages and disadvantages of each methodology used in this study are pointed out. We suggest that this multiplex qPCR can be used as a rapid screening technique for the analysis of food microbiological quality.

## 1. Introduction

Foodborne diseases (FBDs) constitute a serious public health problem worldwide, owing to the significant morbidity and mortality rates associated with FBDs. The Centers for Disease Control and Prevention (CDC) estimates that, each year, approximately 48 million Americans are infected, 128 000 are hospitalized, and 3000 die from FBDs [1].* Salmonella *spp.,* Escherichia coli*, and* Staphylococcus aureus *are among the ten most common bacteria causing notified bacterial FBD globally [2] and are also in the list of the main causes of diseases, hospitalizations, and deaths from FBD in the United States [3] and in Brazil [4]. In 2016, the CDC estimated the number of illnesses, hospitalizations, and deaths from FBD in the United States;* Salmonella* spp. (nontyphoid) and* S. aureus *were among the prevailing pathogens related to illnesses, holding the second and fifth places, respectively. Concerning hospitalizations, infections by* Salmonella* and* E. coli *(STEC 0157) occupied the first and fifth places, respectively, and, among the FBDs resulting in death,* Salmonella* occupied the first place [3]. In Brazil, research carried out between the years 2000 and 2016 confirmed that, among the 11.477 notified outbreaks of FBDs, 1627 were caused by* Salmonella* spp. (14.2% of the total), 865 by* S*.* aureus* (7.5%), and 749 by* E. coli* (6.5%) [4, 5]. However, the true incidence is difficult to determine owing to subnotification and nonidentification of the cause of the outbreaks.

In addition to providing information on the epidemiological relevance of pathogens in FBDs, the quantification of these pathogens in foods can provide information about feedstock quality and about the possible failures during food processing. For example, the presence of these microorganisms can indicate fecal contamination of human or animal origin (*E. coli*) and presence of pathogens (*Salmonella *spp.) and can further indicate inadequate sanitary conditions during the processing of the product (*S. aureus*). Thus, methods that rapidly quantify these pathogens in real time can be used as a tool for quality management focused on food safety. Currently, food safety not only is a concern to public health, but also corresponds to a competitive advantage in the food industries, because a consumer who is more interested and concerned about the quality of consumed products presses the market to offer quality products and services [9].

In recent years, the food industries have adopted methods used as microbiological quality management tools for the rapid detection of FBD-causing microorganisms and deteriorating organisms [10]. To obtain quick results and enable the handling of several samples in the same analysis, various methods have been developed in recent decades, comprised of many different detection technologies based on culture with differential plating media, serological, and molecular techniques. Among them, the quantitative polymerase chain reaction (qPCR) is a sensitive method that quantifies the number of pathogens in a sample through the quantification of bacterial DNA in real time. When compared to other tests used for microbial contamination analysis in foods, qPCR is considered more sensitive and specific. Furthermore, through this method, it is possible to perform multiplex testing, allowing the simultaneous quantification of more than one pathogen in a single reaction [11, 12], thus making it an important tool for food analysis.

Thus, this study aimed to standardize the qPCR technique for the simultaneous quantification of* Salmonella *spp.,* E. coli*, and* S. aureus*, through the development of a multiplex test, thus proposing its use for food analysis. This methodology was applied in different food matrices (milk, beef, and oyster meat), and the results were compared with microbiological culture methodologies, such as the official culture method (performed according to the Brazilian legislation) and the ready-to-use test Compact Dry.

## 2. Materials and Methods

### 2.1. Bacterial Strains

Strains of* Salmonella enterica *serovar Enteritidis PT4,* E. coli* (INCQS 00033), and* S. aureus *(INCQS 00186) obtained from the microbial culture collection of the National Institute of Health Quality Control (INCQS, Instituto Nacional de Controle de Qualidade em Saúde) of Oswaldo Cruz Foundation/RJ were used in this study. An isolated colony of each microorganism was inoculated in 1.0 mL of tryptic soy broth (TSB; HiMedia, Mumbai, India) and incubated at 37°C for 18–24 h. This bacterial suspension was then used for genomic DNA extraction using the Easy DNA Extraction Kit (Invitrogen, Carlsbad, CA, USA). The DNA from each strain was quantified at 260 and 280 nm using the Nano Drop 2000 spectrophotometer (Thermo Scientific, Waltham, MA, USA) and was then used in conventional PCR to amplify the target gene of each strain and produce the qPCR standard curve.

### 2.2. Amplification of Target Genes

A conventional PCR was performed using specific primers for the target genes of each bacterium (Table 1) in individual reactions. The amplifications were performed in a final volume of 50 *μ*L containing 0.2 *μ*M of each primer (forward and reverse, Table 1), 0.2 mM dNTPs, 1.5 *μ*M MgCl_2,_ 2.0 U Taq DNA Polymerase (Invitrogen), PCR 1X buffer, and 3 *μ*L of DNA. Sterile ultrapure water (DNase- and RNase-free) was added to reach a final reaction volume of 50 *μ*L. The reactions were performed in a Proflex PCR thermal cycler system (Applied Biosystems, Life Technologies, Carlsbad, CA, USA) using the following program: one cycle of 94°C for 5 min, 32 cycles of 94°C for 60 s, 58°C for 30 s, and 72°C for 60 s, and one cycle of 72°C for 10 min. The PCR products were visualized after 1% agarose gel electrophoresis by staining with Sybr Safe (Invitrogen). Subsequently, the PCR products were purified using a PureLink™ Quick Extraction Kit (Invitrogen) and quantified using the Nano Drop 2000 spectrophotometer (Thermo Scientific).

### 2.3. Production of Standard Curves

The purified products of target gene amplifications for each bacterial strain were diluted to 20 ng/*μ*L and the gene copy numbers were determined by the formula:

  Gene copy number = Amount of DNA (*μ*g) × 6.022 × 10^23^/DNA fragment (bp) × 10^6^ × 650.

Standard curves used in qPCR were built using serial dilutions (10X) of the target genes from each strain, as follows:* ssf Salmonella* (8.64 × 10^1^ to 8.64 × 10^6^ copies),* pho*A* E. coli* (7.2 × 10^1^ to 7.2 × 10^5^ copies), and* nuc S*.* aureus* (1.3 × 10^1^ to 1.3 × 10^6^ copies).

### 2.4. Multiplex qPCR for Simultaneous Quantification of* Salmonella *spp.,* E. coli*, and* S. aureus*

Multiplex qPCR was performed using TaqMan Fast Advanced Master Mix (Invitrogen) for the simultaneous quantification of the three pathogens.

The multiplex qPCR was performed in an AB 7500 Fast (Applied Biosystems) using TaqMan. MGB probes and primers (Table 1) were designed using software Primer Express, version 3.0 (Life Technologies).

Amplifications were performed at a final volume of 20 *μ*L containing 2.0 *μ*L of DNA corresponding to each point of the curve, 10.0 *μ*L of TaqMan Fast Advanced Master Mix reagent (Invitrogen), and forward and reverse primers at a concentration of 5 *μ*M. For the* Salmonella* and* E. coli* amplifications, 0.5 *μ*L of specific primers was used in each reaction, and for the* S. aureus* amplification, 0.4 *μ*L of primers was used. In addition, 0.5 *μ*L of each MGB TaqMan probe specific for* Salmonella* (FAM) and* E. coli* (NED) and 0.4 MGB TaqMan probe for* S. aureus* (VIC) were used at a concentration of 5 *μ*M. Sterile ultrapure water (DNase- and RNase-free) was then added to reach a final volume of 20 *μ*L. Each run consisted of one cycle at 50°C for 2 min, one cycle at 95°C for 20 s, and 45 cycles at 95°C for 3 s and 60°C for 30 s.

### 2.5. Sensitivity, Specificity, and Reproducibility of qPCR

To evaluate intra- and interassay reproducibility, the average of the cycle threshold (C_T_), the standard deviation (C_T_ SD), and the coefficient of variation (CV) were calculated in five different reactions, including three replicates of each target gene, using known concentrations of 10^5^ to 10^1^ copies of each target gene.

The limit of detection of each gene was determined using 1:2 serial dilutions as follows:* ssf Salmonella *(864, 432, 216, 108, 54, 27, 13.5, and 6.75 gene copy numbers),* pho*A* E. coli *(396, 198, 99, 41.5, 24.75, 12.38, and 6.19 gene copy numbers), and* nuc S. aureus *(671, 355.5, 167.75, 83.87, 41.93, 20.96, 10.48, and 5.24 gene copy numbers). In order to confirm the specificity of the primers and probes used in qPCR, the sequences of target genes were initially aligned using the Basic Local Alignment Search Tool (BLASTn) (http://blast.ncbi.nlm.gov/Blast.cgi) to check the similarity with sequences available in the database.

### 2.6. Determination of Gene Copy Number in a Single Bacterial Colony-Forming Unit (cfu)

The bacterial strains were inoculated on tryptic soy agar plates (TSA; HiMedia) and incubated at 37°C for 18–24 h. After this period, a colony of each bacterium was used for the TaqMan qPCR, as described above. The colonies of* Salmonella *and* E. coli *were used directly in the reaction. For* S. aureus, *one colony was first transferred to a microtube containing 10 *μ*L of sterile ultrapure water and subjected to heating at 100°C for approximately 15 min in a dry water bath (Loccus Biotecnologia, Cotia, SP, Brazil) until all the water evaporated; the remaining content was used in the reaction. The experiments were carried out in triplicate.

### 2.7. Application of Multiplex qPCR Technique and Microbiological Culture Methodologies for* Salmonella *spp.,* E. coli*, and* S. aureus* Quantification in Different Food Matrices

**Figure 1:**
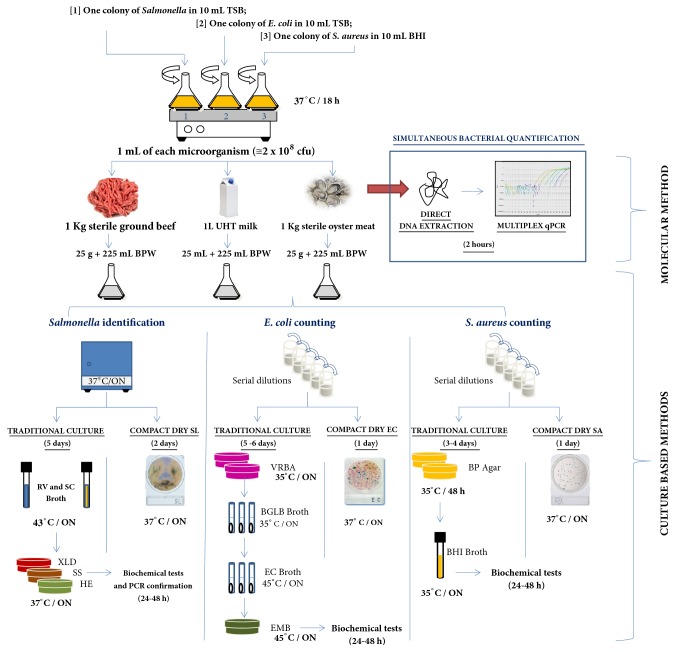
Schematic summary of the methodological procedure for artificial bacterial inoculation in different food matrices and comparison of multiplex qPCR technique with microbiological culture methodologies for* Salmonella* spp. detection,* Escherichia coli*, and* Staphylococcus aureus* quantification (see Material and Methods).

Three different food matrices (ground beef, milk, and oyster meat) were used to compare multiplex qPCR technique with microbiological culture methodologies, such as the official culture method (performed according to the Brazilian legislation) and the rapid test Compact Dry® (HyServer), for* Salmonella *spp.*, Escherichia coli*, and* Staphylococcus aureus* quantifications.  Figure 1 shows a schematic summary of the methodological procedure.

One colony of* Salmonella enterica* serovar Enteritidis phage type 4 and* Escherichia coli* were inoculated separately in 10 mL TSB (HiMedia) and one colony of* Staphylococcus aureus* was inoculated in 10 mL Brain Heart Broth (BHI; HiMedia). The bacterial suspensions were incubated at 37°C/18 h under constant agitation (130 rpm). One mL of each culture (approximately 5 × 10^8^ cfu) was inoculated together in each food matrix (1 Kg sterile ground beef, 1L UHT milk, and 1 Kg sterile oyster meat) and then, the food was homogenized for 5 min in a tissue mixer (Novatecnica, Brazil). For multiplex qPCR analyses, 1.0 g of each sample food was used for DNA extraction using the Easy DNA extraction Kit (Invitrogen). The DNA samples were quantified using the Nano Drop 2000 (Thermo Scientific) and were diluted to 50 ng/*μ*L. The DNA (2 *μ*L) was used to estimate gene copy numbers for each bacterial strain through qPCR, using TaqMan multiplex reactions, as described previously.

For microbiological culture analyses, 25 g (or 25 mL) of each food homogenate was mixed with 225 mL of 0.1% peptone water (Acumedia). The mixtures were homogenized again and 10-fold serially diluted in triplicate. The samples were analyzed using rapid identification kits (Compact Dry®, HyServe GmbH & Co. KG, Uffing, Germany), according to the manufacturer's instructions, to enumerate total coliforms and* Escherichia coli *(Compact Dry EC) and* Staphylococcus aureus* (Compact Dry XSA) and detect* Salmonella* spp. (Compact Dry SL). Analyses were also performed according to the Brazilian legislation described in the Normative Instruction No. 62 of August 26, 2003, of the Ministry of Agriculture, Livestock and Supply that addressed the Official Analytical Methods for Microbiological Analysis of Products of Animal Origin and of Water [13] that is in accordance with “Compendium of Methods for the Microbiological Examination of Foods” of American Public Health Association (APHA), as described below.

*Enumeration of Coliforms and E. coli*. The total coliform and* E. coli* counts were determined by plating the samples on solid medium. Aliquots (1 mL) of each dilution were cultured on violet red bile agar (VRBA; HiMedia) and the plates were incubated at 35°C for 18–24 h. Five presumptive colonies were picked and each was transferred to a tube containing brilliant green lactose broth (BGLB; HiMedia), and incubated at 35°C. The tubes were examined at 24 and 48 h for gas production and to determine the coliform count at 35°C. One aliquot of each gas-positive tube was cultured in EC broth (HiMedia) and incubated at 45°C. The tubes were also examined at 24 h for gas production and to determine the coliform count at 45°C. One aliquot of each gas-positive tube was cultured in eosin methylene blue agar (EMB; HiMedia) and incubated at 45°C for 24 h. The suspect colonies were counted and tested by specific biochemical analysis (indole, methyl red, Voges-Proskauer, and Simon citrate test) to confirm the presence of* E. coli*. 

*Enumeration of Staphylococcus aureus*. One milliliter of each dilution was divided on the surface of three Baird-Parker (BP; Acumedia Neogen do Brasil, Indaiatuba, SP, Brazil) agar plates. The plates were incubated at 35°C for 48 h and five presumptive colonies were selected for catalase, coagulase, and thermostable DNase tests. 

*Detection of Salmonella spp*. For detection of* Salmonella *spp., 25 g of the sample was mixed with 225 mL of buffered peptone water and incubated at 37°C. After 24 h, 1 mL was transferred from each tube to 9 mL selenite-cystine (SC; Merck KGaA, Darmstadt, Germany) broth and Rappaport-Vassiliadis (RV; Merck) broth and incubated at 43°C for 24 h. A sample (1 mL) from each broth was plated onto xylose-lysine deoxycholate (XLD; Acumedia), Hektoen Enteric (HE; HiMedia), and* Salmonella*-*Shigella* (SS; Merck) agars. The plates were incubated overnight at 37°C. Typical colonies were submitted to biochemical screening on triple sugar iron agar (TSI; HiMedia), lysine iron agar (LIA; HiMedia), and urea agar (UA; Merck). The presence of* Salmonella *spp. was confirmed by testing presumptive colonies using two sets of primers to amplify a conserved region for* Salmonella* genus: ST11 (5′-AGCCAACCATTGCTAAATTGGCGCA-3′) and ST15 (5′-TTTGCGACTATCAGGTTACCGTGG-3′) [1]. The 25 *μ*l PCR mixture contained 1X PCR buffer (Invitrogen), 1.25 mM MgCl_2_, 200 *μ*M each deoxyribonucleoside triphosphate (Invitrogen), 10 pmol sense and anti-sense primers (Invitrogen), 1.25 U Taq DNA polymerase (Invitrogen), and one suspected* Salmonella *colony. The volume of the reaction mixture was made up with ultrapure water. The amplification cycle consisted of an initial denaturation step at 94°C for 5 min, followed by 35 cycles of 94°C for 30 s, 60°C for 30 s, and 72°C for 1 min, and a final extension step at 72°C for 10 min. The PCR products were visualized by loading 5 *μ*L suspension onto 1% agarose gel, staining with SYBR® Safe (Invitrogen), and examining the same under UV light.

### 2.8. Statistical Analysis

The average and standard deviations of bacterial quantities detected by all tests were calculated, submitted to variance analysis (one-way ANOVA), and compared by Tukey's test. For the comparison of variances, Bartlett's test was used. The values of p ≤ 0.05 were considered statistically significant. Data were analyzed using the Software GraphPad Prism, version 5.03 (San Diego, CA, USA).

## 3. Results

### 3.1. Standard Curves

**Figure 2:**
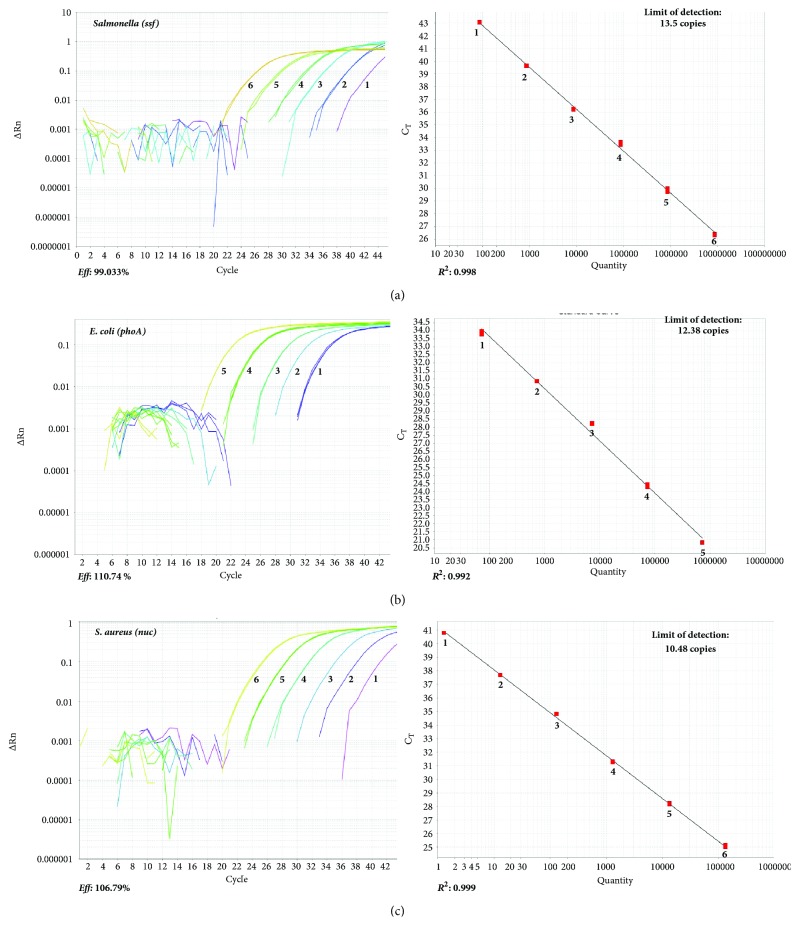
Amplification curves (left) and standard curves through TaqMan qPCR of serial dilutions of target genes. Copy numbers of each gene: (a)* ssf *from* Salmonella *spp. (8.64 × 10^6^ to 8.64 × 10^1^); (b)* pho*A from* Escherichia coli* (7.2 × 10^5^ to 7.2 × 10^1^); (c)* nuc *from* Staphylococcus aureus* (1.3 × 10^5^ to 1.3 × 10^0^).

In qPCR reactions, the linear correlation coefficient (*R* ^2^) of the standard curves of the three microorganisms was high: 0.998 for* Salmonella*, 0.992 for* E. coli*, and 0.999* S. aureus*. The amplification curve presented an* Eff *of 99.033% for* Salmonella*, 106.79% for* S. aureus*, and 110.74% for* E. coli *(Figure 2).

### 3.2. Sensitivity, Specificity, and Reproducibility of qPCR

In qPCR reactions, the limit of detection was 13, 10, and 12 copies for* ssf* (*Salmonella*)*, pho*A (*E. coli*), and* nuc *(*S. aureus*) genes, respectively.

Through the BLASTn Program, all sequences amplified by the primers described in this study showed 100% similarity with* Salmonella *(AE 006468.2),* E. coli *(FJ546461), and* S. aureus *(AP 017320.1).

The coefficients of variation (CV) of the intra- and interassays were statistically low. The CV of the interassay was 0.41% for* Salmonella*, 0.19% for* E. coli*, and 0.15% for* S. aureus* (Table 2). For the intra-assay, the CV was 1.03% for* Salmonella, *2.8% for* E. coli*, and 2.5% for* S. aureus* (Table 2).

### 3.3. Determination of Gene Copy Numbers in One Bacterial Colony-Forming Unit (cfu)

Quantification of the* nuc* gene in one cfu of* S. aureus* showed there were 7.9 × 10^11^ copies/ cfu. The* phoA* gene was present in 1.28 × 10^7^ copies/ cfu in* E. coli*, and* ssf* was present in 2.10 × 10^8^ copies/ cfu in* Salmonella*. The CV between triplicates was less than 1.4% in all amplifications (Table 3).

### 3.4. Comparison between Multiplex qPCR and Microbiological Culture Methodologies for* Salmonella *spp. Detection,* E. coli*, and* S. aureus* Quantification in Different Food Matrices

**Figure 3:**
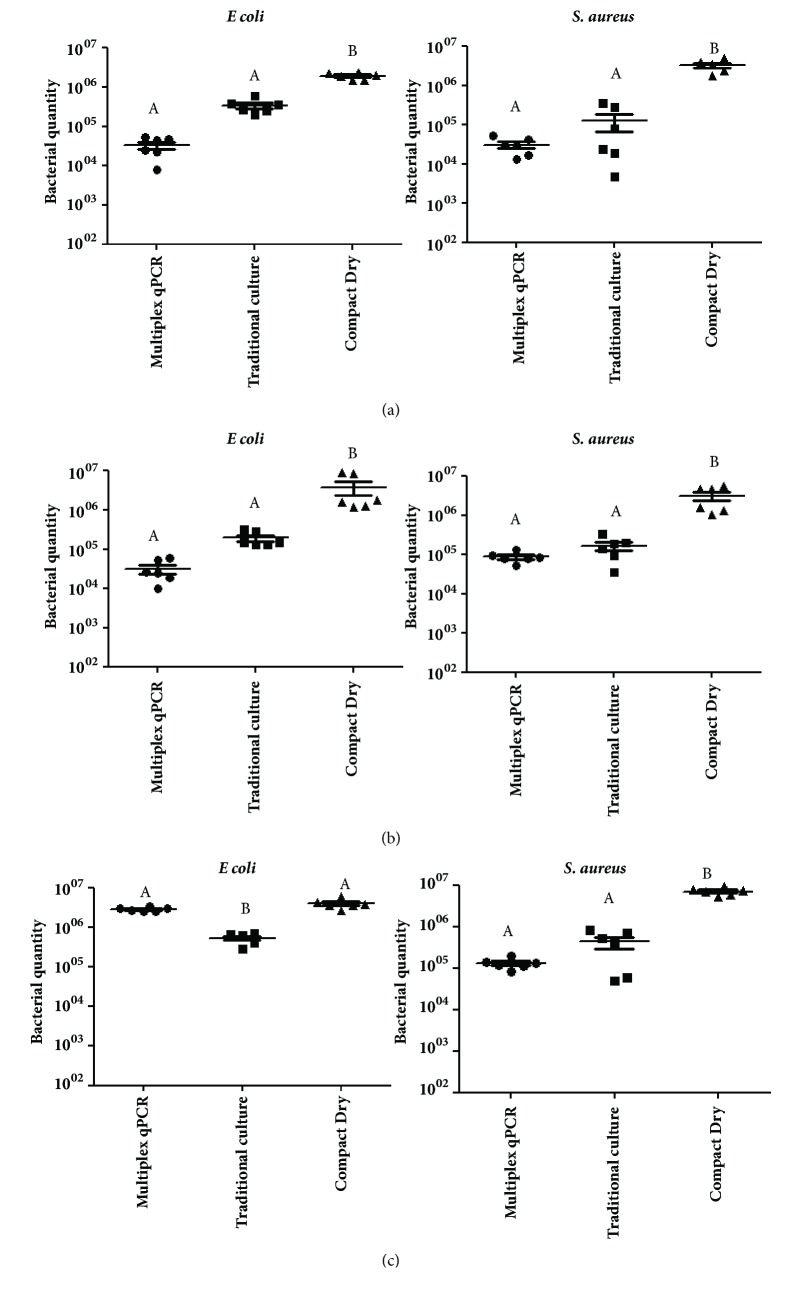
Average between* Escherichia coli *(in the left) and* Staphylococcus aureus *(in the right) quantities detected by multiplex qPCR assay, traditional culture method, and Compact Dry after artificial bacterial inoculation in UHT milk (a), sterile ground beef (b), and sterile oyster meat (c). In qPCR, the gene copy numbers (*phoA* for* E. coli* and* nuc* for* S. aureus*) determined the bacterial quantities. In traditional culture and Compact Dry methodologies, colony-forming unit (cfu/g or cfu/mL) determined the bacterial quantities in food. Different letters mean statistical difference by Tukey's test (p ≤ 0.05).

No statistically significant difference was observed in the comparison between the averages of* E. coli *and* S. aureus* quantities detected by multiplex qPCR and traditional culture in milk and ground beef samples, although the difference in approximately one log in bacterial quantity was detected. In these food matrices, both tests presented significant difference when compared with Compact Dry (Figures 3(a) and 3(b)). The same was observed for* S. aureus *quantification in oyster meat (Figure 3(c), right). However, for* E. coli* quantification in this food matrix, the traditional culture showed significant difference when compared with multiplex qPCR and Compact Dry (Figure 3(c), left).

For* Salmonella* spp. quantification through multiplex qPCR, the averages of the* ssf* copy numbers were 5 log_10_, 5.1 log_10_, and 4.8 log_10_ in milk, ground beef, and oyster meat samples, respectively. These results could not be compared with culture methodologies because those methods are not used to quantify this pathogen but only to detect it.

## 4. Discussion

For food quality control, the standardization of methods that simultaneously quantify the three main foodborne pathogens (*Salmonella* spp.,* E. coli*, and* S. aureus*) generates fast results that allow the early intervention of control strategies. It can also be an important tool for quantitative microbial risk assessment, which requires numerical data that evaluate the performance objectives in a productive chain, determining the level of contamination at a specific stage of food production, and evaluating if the hazard is diminished (or eliminated) after processing or after control measures [14]. Thus, qPCR using probes marked with fluorophores that emit fluorescence at different wavelengths can be a good alternative for use as a rapid test; it allows the amplified products of two or more regions of DNA to be quantified in a specific manner for specific targets in the same reaction, providing results in real time [15].

The sensitivity, amplification efficiency, reproducibility, and coefficient of linearity of the standard curves in qPCR were found to be consistent. The combination of primers and probes designed in this study retained the expected efficiency in multiplex analysis for the simultaneous quantification of* Salmonella*,* E. coli*, and* S. aureus*. The amplification efficiency (*Eff* %) assesses whether the primer pairs amplify the target gene exponentially at each cycle and must be between 90 and 110%. The reactions with* Eff* within these values are considered efficient [16]. The standard curves for* Salmonella*,* E. coli*, and* S. aureus* quantifications were highly reproducible, as indicated by the low intraexperiment (< 6.0%) and interexperiment (< 1.0%) CV (Table 2). A good linear correlation was also obtained in all curves (> 0.99).

Multiplex qPCR reaction demonstrated high sensitivity for enumerating small amounts of DNA molecules. This can be confirmed by the limit of detection of 13 copies for* ssf *(*Salmonella), *10 copies for* pho*A gene (*E. coli*), and 12 copies for* nuc *gene (*S. aureus*). Usually, researchers evaluate the limit of detection of the qPCR techniques by counting cfu/g or cfu/mL, so they can determine the minimal amount of cfu in food that can be detected by qPCR. According to previous studies, the limit of detection of* Salmonella *in food was 2 to 5 cfu/25 g and 5 cfu/100 g [17, 18]. For* E. coli, *the limit of detection has been described as 1 to 5 cfu/25 g [19, 20] and for* S. aureus, *Elizaquiável and Aznar [21] could detect 10^3^ cfu/g by qPCR. These studies did not determine the gene copy numbers* per* cfu, because the tests were qualitative with the objective of pathogen detection and not pathogen quantification. In our work, since the objective was pathogen quantification, the determination of gene copy numbers in one cfu was necessary, mainly because we did not use any methodology to enrich the food samples; therefore, we could predict the contamination level of the food earlier, even before the bacteria grew to form colonies. We assumed that if we could determine the average gene copy number in one cfu, the quantitative results generated by qPCR could provide data that allow us to infer the level of food contamination per bacterial cells. However, the qPCR does not define the viability of bacterial cells, because the gene can be detected even in unviable cells [22]. The determination of the gene copy numbers in a single cfu of* Salmonella, E. coli*, and* S. aureus* using TaqMan showed a low CV in repetitions (average 0.77 ± 0.5, Table 3), which demonstrates high repeatability. One cfu of* S. aureus* produced 7.9 × 10^11^ *nuc* gene copies, showing three to four logs more gene copies than* ssf *in* Salmonella *(2.10 × 10^8^) and* pho*A in* E. coli* (1.28 × 10^7^), respectively. This difference must be considered during the multiplex analysis, because the determination of increased copy numbers of* nuc* gene does not mean that the food is more contaminated with* S. aureus* than with* E. coli* or* Salmonella*.

The average of bacterial quantification in the different food matrices through multiplex qPCR was 5.7 log_10_, and no statistical difference was observed compared with traditional culture methodology (5.5.log_10_). Despite this, in milk and ground beef, approximately one log_10_ of difference was observed (Figure 3). This result can be caused by competition of primers for the reagents available in the reaction mix, since there is no concentration's variation of its components, as the mix is ready to use (according to manufactory's instruction). This means that the same mix used for singleplex reactions is used the same way in multiplex reactions, probably reflecting the competitive nature of the process. In addition, the amplification of one target DNA (including nonspecific products) may be more expressive than the other targets, resulting in a decrease of the efficiency and sensitivity in multiplex reaction [23]. This difficulty in performing multiplex tests is described as one of the disadvantages of real-time PCR, including other points, such as the need for qualified personnel, the high cost of equipment, and its inherent ability to not distinguish living cells and dead cells [24]. However, the authors also emphasize the advantages of using this molecular technique for diagnosis; since it can be monitored in real time, it does not need to perform any postreaction processing, such as the electrophoresis gel; the reactions are rapid due to short cycles, confirmation of amplification in real time, and being specific, sensitive, and reproducible reactions. Thus, multiplex qPCR can be a powerful tool for fast screening of large number of samples. In addition, for* Salmonella* diagnosis, different from culture methods, qPCR allow enumeration of the pathogen, being a useful tool for Quantitative Microbial Risk Assessment, in which quantitative data are recommended [22].

The average of bacterial quantification in the different food matrices through the ready-to-use test Compact Dry was 6.6 log_10_, presenting significant difference when compared with traditional culture method and multiplex qPCR. In our study, this method presented high sensitivity, detecting one log_10_ more than the bacterial amounts inoculated in the food, increasing the numbers of false-positive samples. Differently, previous studies performed by Teramura [25], Hosokawa [26], and Kodaka [27] obtained compatible results of this chromogenic method when compared to traditional culture techniques. For food industries, the advantages of this method include ease of sample inoculation, smaller size than conventional plates, being easy to discard [26], reduction of practical use and laboratory time, less employee training, longer shelf life, storage space [27], being an easy screening method for bacterial enumeration, and useful for quality control.

The traditional culture methodology performed in this study obtained results close to the bacterial amounts inoculated in food. Jasson [28] describes that this standardized method of classical culture is still in use by many laboratories, especially by regulatory agencies, because they are harmonized methods, considered as the “gold standard” in food diagnostics. However, the disadvantage is that although they do not require expensive infrastructure, laboratories must be equipped, analyses are labor-intensive to execute, require the use of large volumes reagent media, and encompass procedures that take so long in the analysis as in the data collection.

Each technique has its particularity and the purpose of use depends on objective, infrastructure, and time available to obtain results.  Table 4 summarizes each method used in this study, pointing out the advantages and disadvantages, and purposes of use in food industries.

## 5. Conclusion

The technique described in this study can be tested for use in simultaneously quantifying* Salmonella, E. coli*, and* S. aureus *at different stages of production/processing in the food industries, in order to assess whether microbiological hazards decrease or increase during the processing steps. By generating specific results related to the quantities of each microorganism, the increased copy numbers of a target gene can provide information about the type of contamination that may be occurring in a processing step. For example, increased copy numbers of* nuc *gene (*S. aureus*) might imply contamination by handling, increased copy numbers of* pho*A gene (*E. coli*) might suggest fecal contamination, and increased copy numbers of* ssf* (*Salmonella*) might indicate that the processing has not been able to eliminate pathogenic microorganisms. This approach would aid in achieving more targeted quality control.

## Figures and Tables

**Table 1:** Primers and probes used in PCR and qPCR.

Reference strain	Target gene (GenBank accession no.)	Primes 1 (5′-3′)^a^	bp	Reference	Primers 2 (5′-3′) and Probes^b^	bp
*Escherichia coli* ATCC 25922 INCQS 00033	*phoA* (FJ546461)	F:GGTAACGTTTCTACCGCAGAGTTG	468	Shome et al. 2011 [6]	F: CCGGGTAACGCTCTGGAA	54
		R:CAGGGTTGGTACACTGTCATTACG			R: AAGCAGCTGTTCGGTAATCGA	
					P:AAGGCGGAAAAGG	

*Salmonella* Enteritidis PT4 (IOC)	*Salmonela specific fragment*: (*ssf) *patent EP0707659 A1	F (ST15): GGTAGAAATTCCCAGCGGGTACTG	429	Aabo et al. 1993 [7]	F: CGGCGAATTTTTGCGACTAT	59
					R: TGGCTTCGCTTTATGTTCGA	
	(AE006468.1:fragment 1409127 to 1409555)	R (ST11): AGCCAACCATTGCTAAATTGGCGCA			P: AGGTTACCGTGGAGGC	

*S. aureus* ATCC 6538 INCQS 00186	*nuc *: nuclease (NC_002758.2)	F: GCGATTGATGGTGATACGGTT	276	Brakstad et al., 1992 [8]	F:GGTCAACCAATGACATTCAGACTATT	82
		R: CAAGCCTTGACGAACTAAAGC			R: GCCATATTTCTCTACACCTTTTTTAG	
					P: TGATACACCTGAAACAAA	

^a^Used in the conventional PCR to amplify the target gene. The PCR product was used to build the standard curve of the qPCR.

^b^Designed in this study through Primer Express® Software For Real-Time PCR, version 3.0 (Applied Biosystems™).

**Table 2:** Inter- and intra-assay reproducibility of qPCR.

Gene copy numbers	Intra-assay Reproducibility^a^			Interassay Reproducibility^b^		
	Ct (average)	SD	CV (%)	Ct (average)	SD	CV (%)
*Salmonella (ssf)*						

8.62 × 10^6^	26.86	0.58	2.10	26.36	0.06	0.20
8.62 × 10^5^	30.29	0.53	1.00	29.86	0.20	0.60
8.62 × 10^4^	33.83	0.35	1.00	33.53	0.17	0.50
8.62 × 10^3^	36.47	0.29	0.80	36.22	0.04	0.10
8.62 × 10^2^	39.71	0.13	0.30	39.62	0.02	0.05
8.62 × 10^1^	42.54	0.70	1.00	42.54	0.70	1.00

*Escherichia coli (phoA)*						

7.92 × 10^5^	21.67	0.97	4.00	20.83	0.02	0.10
7.92 × 10^4^	24.99	0.80	3.00	24.29	0.001	0.01
7.92 × 10^3^	29.00	0.89	3.00	28.23	0.03	0.10
7.92 × 10^2^	31.65	1.08	3.00	30.72	0.21	0.70
7.92 × 10^1^	34.39	0.47	1.00	33.97	0.001	0.02

*Staphylococcus aureus (nuc)*						

1.30 × 10^5^	25.67	1.93	6.00	25.08	0.10	0.40
1.30 × 10^4^	28.83	1.44	4.00	28.19	0.04	0.10
1.30 × 10^3^	31.66	0.62	1.00	31.29	0.03	0.10
1.30 × 10^2^	34.70	0.37	1.00	34.79	0.05	0.10
1.30 × 10^1^	37.59	0.16	0.40	40.80	0.14	0.03

^a^Average between three replicates.

^b^Average between five different reactions.

Ct: cycle threshold; SD: standard deviation; CV: coefficient of variation.

**Table 3:** Specific gene copy numbers in one colony-forming unit (cfu) through TaqMan qPCR.

Microorganism (target gene)	Ct^a^	SD	CV (%)	Gene copy number/cfu^a^
*Salmonella (ssf)*	58.78	0.20	0.34	2.10 × 10^8^
*Escherichia coli (phoA)*	46.94	0.28	0.60	1.28 × 10^7^
*Staphylococcus aureus (nuc)*	47.48	0.64	1.37	7.9 × 10^11^

^a^Average between three replicates.

Ct: cycle threshold; SD: standard deviation; CV: coefficient of variation.

**Table 4:** Advantages, disadvantages, and purposes of use of multiplex qPCR described in this study, ready-to-use Compact Dry, and traditional culture methodology in food industries.

	**Multiplex qPCR**	**Ready-to-use Compact Dry**	**Traditional culture**
**Bacterial amount inoculated**	5.3 log_10_	5.3 log_10_	5.3 log_10_

**Bacterial amount detected (average)** ^**a**^	5.7 log_10_	6.6 log_10_	5.5 log_10_

**Estimated time of analysis**	2 hours (simultaneous quantification of *Salmonella, E. coli and S. aureus*)	1 day (*E. coli and S. aureus*) 2 days (*Salmonella*)	3-4 days ( *S. aureus*) 5-6 days ( *E. coli*) 5 days (*Salmonella*)

**Advantages**	(i) Monitoring in real time; (ii) Does not need to perform post-reaction processing; (iii) Fast; (iv) Confirmation of amplification in real time; (v) Specific, sensitive and reproducible; (vi) Simultaneous quantification of different pathogens.	(i) Ease of sample inoculation; (ii) Smaller size than conventional plates; (iii) Easy to discard (iv) Reduction of practical use and laboratory time; (v) Less employee training;	(i) Standardized method; (ii) “Gold standard” in food diagnostics; (iii) Do not require expensive infrastructure; (iv) Realistic results (similar bacterial quantification to the amount inoculated).

**Disadvantages**	(i) Competitive amplification (decrease of the efficiency and sensitivity in multiplex reaction); (ii) Need for qualified personnel; (iii) High cost of equipment; (iv) Do not distinguish living cells and dead cells.	(i) False positive results; (ii) Spends, at least, one day for results.	(i) Analyses are labor-intensive (ii) Require a lot of reagent media; (iii) Time consuming analysis (more than 3 days).

**Purposes of use**	Fast screening methods of large number of samples. Useful for microbiological quality control.	Screening method for bacterial enumeration. Useful for microbiological quality control.	Official method for food microbiological analysis. Useful for regulatory agencies.

^a^Average of bacterial quantification (*Salmonella, E. coli, *and *S. aureus*) in ground beef, milk, and oyster meat. *Salmonella *was not quantified through Compact Dry and Traditional culture method.

## Data Availability

No data were used to support this study.
